# Retraction Note: Genipin suppression of growth and metastasis in hepatocellular carcinoma through blocking activation of STAT-3

**DOI:** 10.1186/s13046-022-02308-2

**Published:** 2022-03-15

**Authors:** Ming Hong, Selena Lee, Jacob Clayton, Wildman Yake, Jinke Li

**Affiliations:** 1 Science and Technology Innovation Center, Guangzhou University of Chinese Medicine, Guangzhou, China; 2 Institute of Clinical Pharmacology, Guangzhou University of Chinese Medicine, Guangzhou, China; 3 Department of Pharmacology & Toxicology, University of Kansas, Lawrence, KS USA

**Retraction Note: J Exp Clin Cancer Res 39, 146 (2020)**

**https://doi.org/10.1186/s13046-020-01654-3**

The Editor-in-Chief has retracted this article. After publication concerns were raised with respect to the overlap of figures with a previously published article from another author group [1]. Specifically:

- Figure 1b, 1c, 1d, 1e and 1f with Figure 1c, 1d, 1e, 1f and 1h of Figure 1 of [1]
- Figure 2 with Figure 2 of [1]
- Figure 3b and 3c with Figure 3b and 3d of [1]
- Figure 4b, 4c and 4d with Figure 4b, 4c and 4d of [1]
- Figure 5 with Figure 5 of [1]
- Figure 6 with Figure 6 of [1]
- Figure 7 with Figure 7 of [1]

The Editor-in-Chief no longer has confidence in the results and conclusions reported. Ming Hong agrees with this retraction. Jacob Clayton, Wildman Yake and Jinke Li have not responded to correspondence from the Publisher about this retraction. The Publisher was not able to obtain a current email address for Selena Lee.
